# Supplementary material for: Effectors of Puccinia striiformis f. sp. tritici Suppressing the Pathogenic-Associated Molecular Pattern-Triggered Immune Response Were Screened by Transient Expression of Wheat Protoplasts
Source: Int J Mol Sci. 2021 May 7;22(9):4985. doi: 10.3390/ijms22094985 (PMC8125866; doi:10.3390/ijms22094985)

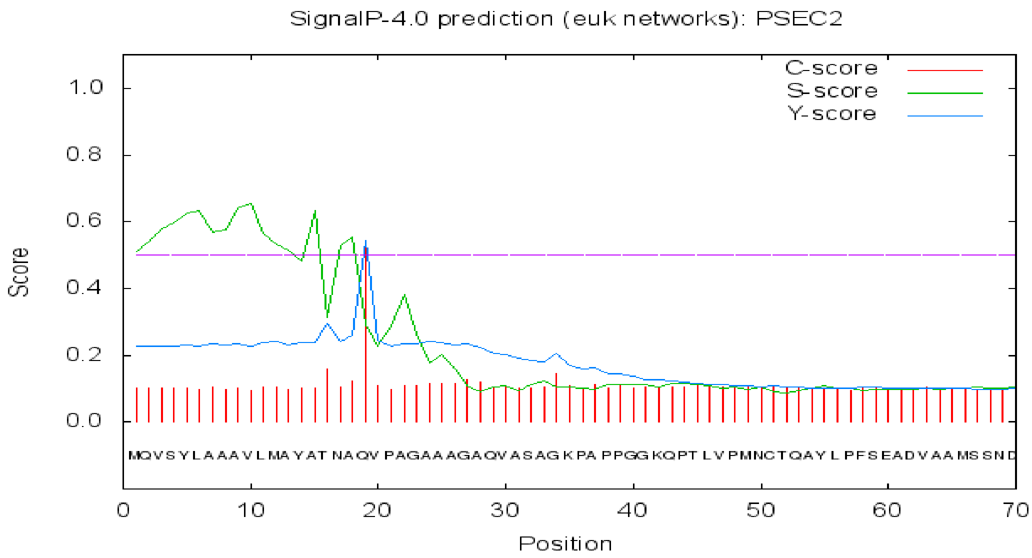
SignalP-4.0 prediction (euk networks): PSEC2
C-score
S-score
Y-score
Score
1.0
0.8
0.6
0.4
0.2
0.0
MQVSYLAAAVLMAYATNAQVPAGAAAGAQVASAGKPAPPGGKQPTLVPMNCTQAYLPFSEADVAAMSSND
0
10
20
30
40
50
60
70
Position

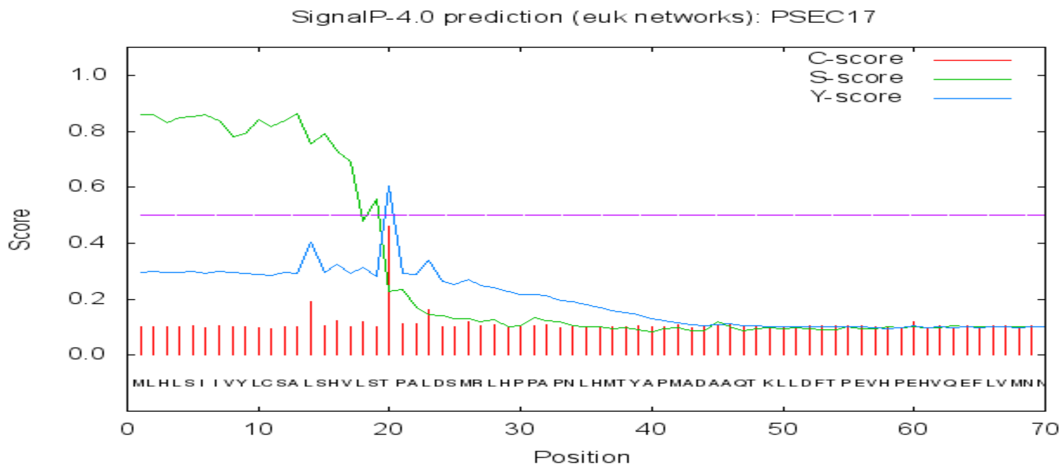
SignalP-4.0 prediction (euk networks): PSEC17
C-score
S-score
Y-score
Score
1.0
0.8
0.6
0.4
0.2
0.0
0
10
20
30
40
50
60
70
Position

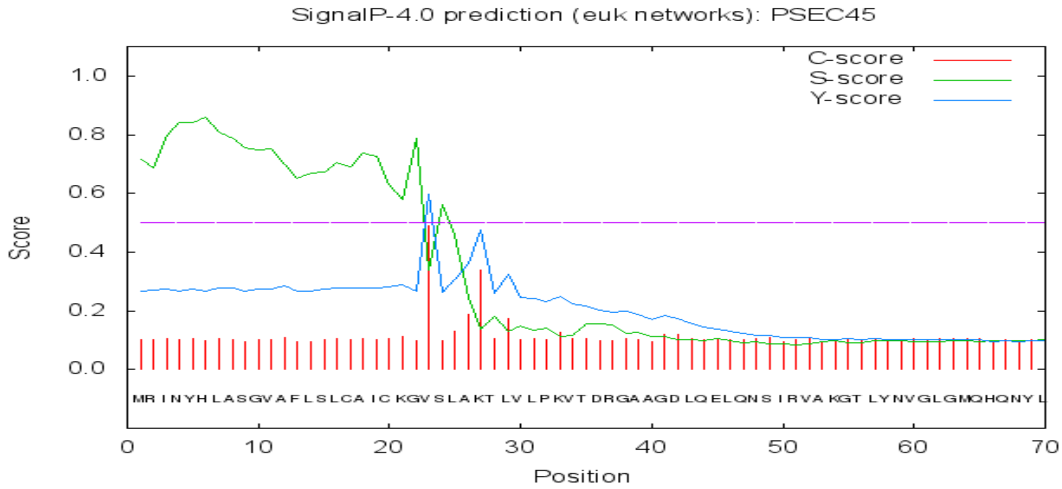
SignalP-4.0 prediction (euk networks): PSEC45
C-score
S-score
Y-score
Score
1.0
0.8
0.6
0.4
0.2
0.0
0
10
20
30
40
50
60
70
Position

TMHMM posterior probabilities for PSEC2

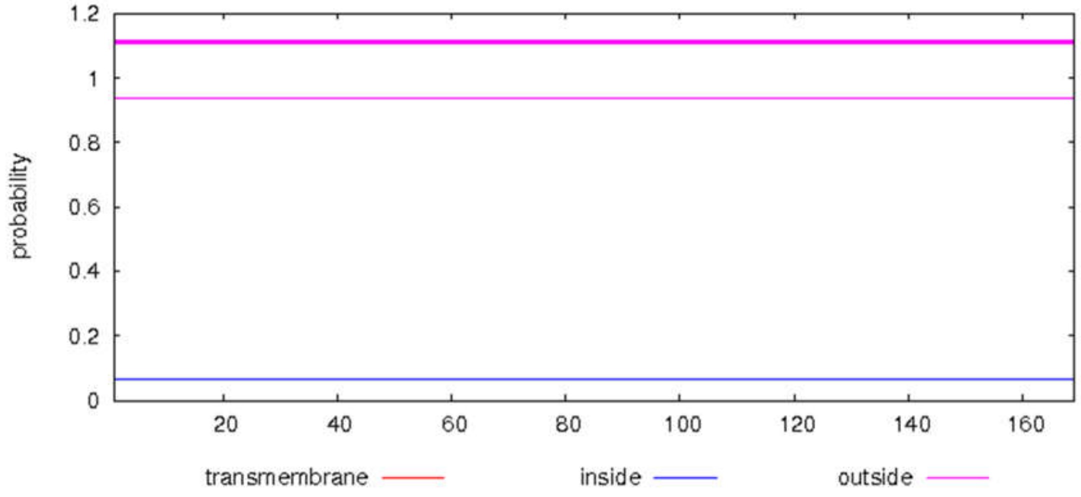


TMHMM posterior probabilities for PSEC17

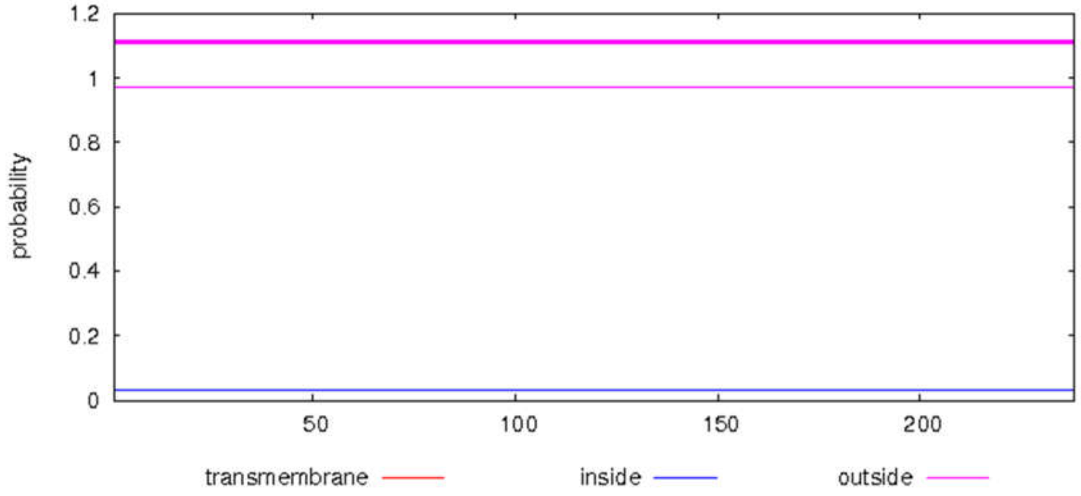


TMHMM posterior probabilities for PSEC45

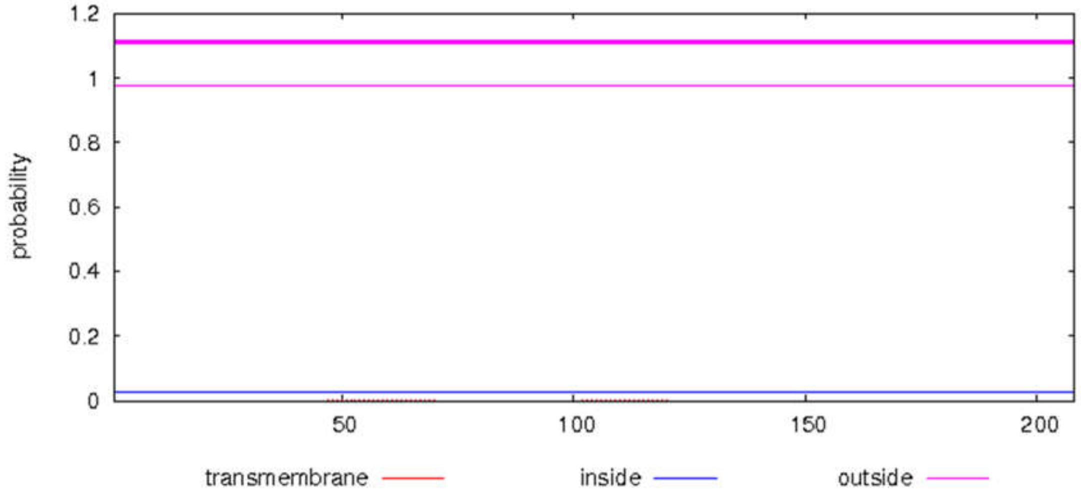

Supplement: Supplementary file 1 [file ijms-22-04985-s001.zip › supporting imformation/Figure S1.pdf]
